# Synthesis of an acyl-acyl carrier protein synthetase inhibitor to study fatty acid recycling

**DOI:** 10.1038/s41598-020-74731-4

**Published:** 2020-10-20

**Authors:** Madeline F. Currie, Dylan M. Persaud, Niralee K. Rana, Amanda J. Platt, Joris Beld, Kara L. Jaremko

**Affiliations:** 1 Department of Chemistry, Hofstra University, Hempstead, NY 11549 USA; 2 Department of Microbiology and Immunology, Drexel University College of Medicine, Philadelphia, PA 19102 USA

**Keywords:** Biochemistry, Chemical biology

## Abstract

Fatty acids are essential to most organisms and are made endogenously by the fatty acid synthase (FAS). FAS is an attractive target for antibiotics and many inhibitors are in clinical development. However, some gram-negative bacteria harbor an enzyme known as the acyl-acyl carrier protein synthetase (AasS), which allows them to scavenge fatty acids from the environment and shuttle them into FAS and ultimately lipids. The ability of AasS to recycle fatty acids may help pathogenic gram-negative bacteria circumvent FAS inhibition. We therefore set out to design and synthesize an inhibitor of AasS and test its effectiveness on an AasS enzyme from *Vibrio harveyi*, the most well studied AasS to date, and from *Vibrio cholerae*, a pathogenic model. The inhibitor C10-AMS [5′-O-(N-decanylsulfamoyl)adenosine], which mimics the tightly bound acyl-AMP reaction intermediate, was able to effectively inhibit AasS catalytic activity in vitro. Additionally, C10-AMS stopped the ability of *Vibrio cholerae* to recycle fatty acids from media and survive when its endogenous FAS was inhibited with cerulenin. C10-AMS can be used to study fatty acid recycling in other bacteria as more AasS enzymes continue to be annotated and provides a platform for potential antibiotic development.

## Introduction

**Figure 1:**
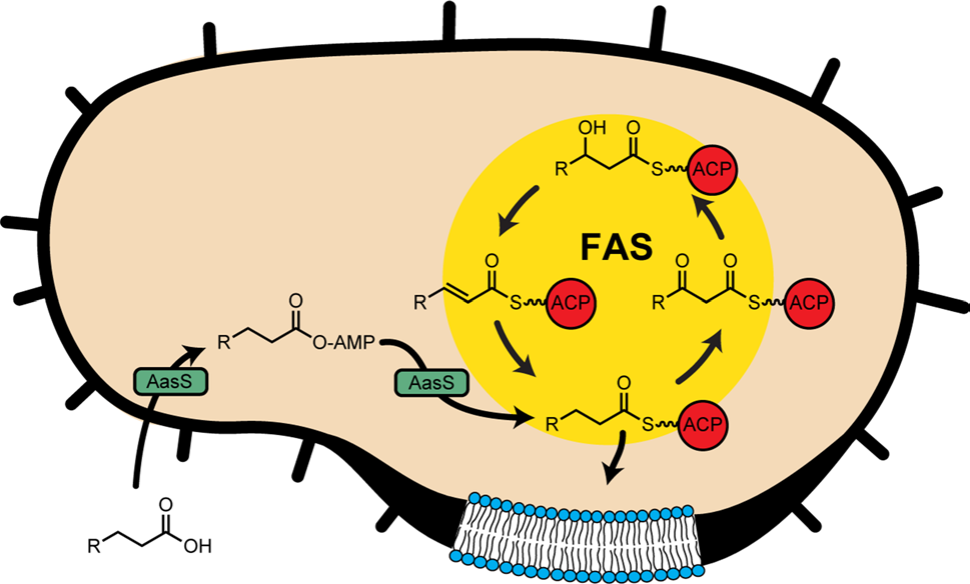
Fatty acid production and recycling in bacteria. The fatty acid synthase (FAS) produces fatty acids endogenously in bacteria in an iterative fashion, and final products get transferred from the acyl carrier protein (ACP) to lipids. AasS can recycle fatty acids from the environment and load them onto ACP in FAS, allowing them to be incorporated into lipids.

Fatty acids have several functions in cells, including energy storage and building lipids used in cell membranes, making them essential to bacteria and most organisms^1^. Fatty acids are obtained by bacteria via two routes: (1) the most common route, endogenous production by a collection of enzymes known as the fatty acid synthase (FAS)^2^, and (2) recycling fatty acids from the surrounding environment^3^. FAS builds fatty acids two carbon units at a time through an iterative series of enzyme reactions^2^. Some gram-negative bacteria can also incorporate exogenous fatty acids into FAS, where they become indistinguishable from the endogenously synthesized fatty acids (Fig. 1)^3^. Central to FAS is the acyl carrier protein (ACP), which is expressed in an inactive *apo* form, but gets post-translationally modified with the addition of a 4′-phosphopantetheine arm attached to a conserved serine residue to make *holo*-ACP. All acyl intermediates are tethered to the 4′-phosphopantetheine arm via a thioester bond while ACP shuttles them to the appropriate enzymes for modification. ACP is essential for all reactions in FAS including initiation, elongation, and transfer to the membrane bilayer (Fig. 1)^2,4^. FASs are classified into type I (FASI), which is characterized by one single multidomain protein and is typical in animals and fungi, and type II (FASII), which employs discrete proteins that form a complex and is typical in bacteria, plants, and cyanobacteria^4^.

**Figure 2:**
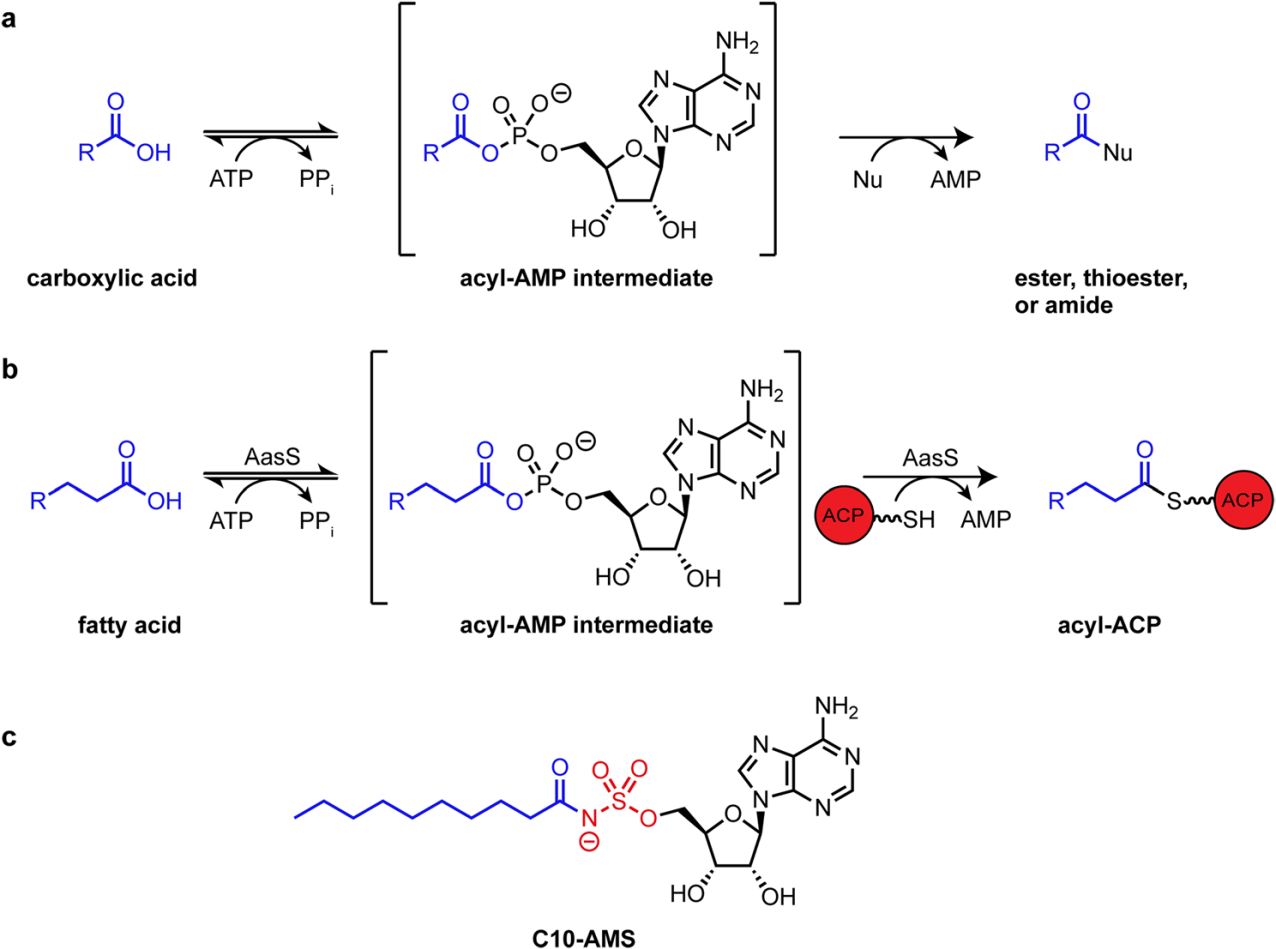
AasS activity and inhibitor design. (**a**) Adenylate forming enzymes catalyze an ATP dependent esterification through a two-step process – activation of a carboxylic acid to make an acyl-AMP intermediate, followed by nucleophilic (Nu) displacement of AMP to form a new bond. (**b**) AasS catalyzes this reaction between a fatty acid and the terminal thiol on ACP. (**c**) The inhibitor, C10-AMS, is designed to mimic the enzymes natural substrate: adenosine in black, the acyl chain in blue, and in red the phosphate group has been replaced with a sulfamoyl moiety.

Gram-negative bacteria can scavenge fatty acids by utilizing an acyl-coenzyme A (CoA) synthetase, which converts exogenous fatty acids into acyl-CoA intermediates that can then be degraded via β-oxidation^3^. Alternatively, a few gram-negative bacteria, cyanobacteria, and plants can utilize an acyl-acyl carrier protein synthetase (AasS) to recycle fatty acids where further degradation is not needed^3^. AasS belongs to a large class of enzymes called adenylate-forming enzymes, which generally catalyze two successive reactions: the activation of an acyl substrate with ATP to form an acyl-AMP phosphodiester intermediate, followed by the attack of this intermediate by a nucleophile to form an ester, thioester, or amide product (Fig. 2a)^5^. Specifically, AasS catalyzes the reaction between a fatty acid and the terminal thiol of *holo-*ACP from FASII (Fig. 2b). Exogenous fatty acids cannot be incorporated into lipids directly, but once loaded onto ACP they are a substrate for lipid biosynthesis. (Fig. 1). AasS enables bacteria to scavenge fatty acids from the environment, potentially helping with survival during high-stress or nutrient limiting conditions^3,6,7^.

As fatty acids are essential for pathogenic bacteria survival, blocking both their endogenous production by FAS and fatty acid recycling is a promising antimicrobial approach. Due to differences between the bacterial type II FAS and the mammalian type I FAS, enzymes in FASII are viable targets for antibiotic therapeutics^8–10^ and there are currently several FASII inhibitors in clinical development^9–13^. Exogenous fatty acids recycled by AasS may enable some gram-negative bacteria to circumvent inhibition of FAS^3,14,15^. Therefore, inhibitors for AasS, to be used in tandem with FASII inhibitors, are needed.

While the first AasS was discovered in *E*. *coli* in 1976, there are currently only five characterized enzymes of this class^7,16–19^. AasSs are not essential to bacteria, similar to many adenylate-forming enzymes involved in secondary metabolite biosynthesis. Nevertheless, there has been a successful anti-mycobacterial program targeting adenylate forming enzymes involved in siderophore biosynthesis, also considered non-essential^20–22^. This highlights the role of accessory pathways that are critical for virulence in vivo and the potential of AasS as an antibacterial target. Adenylate-forming enzymes encompass a diverse group that are involved in many biological processes including protein synthesis, post-translational modification, metabolism, and natural product biosynthesis^23^. Examples include adenylation domains of non-ribosomal peptide synthetases, acyl-CoA ligases, luciferases, and tRNA synthetases. Some enzymes from these classes have been successfully inhibited by small molecules, as they are enticing targets for biological probes and novel therapeutics^24–26^, but AasSs have not been systematically targeted. In this study, we aimed to use these small molecules as a model to design and synthesize an inhibitor for AasS, test the inhibitor’s ability to prevent the loading of fatty acids onto ACP by AasS in vitro, and finally explore the role and inhibition of AasS in fatty acid recycling in vivo.

## Results

### Design and synthesis of C10-AMS

We first set out to design an inhibitor for the acyl-acyl carrier protein synthetase (AasS). AasS catalyzes the ATP-dependent thioesterification of fatty acids to the terminal thiol of the *holo*-acyl carrier protein (ACP). The enzyme works in two steps, going through an acyl-AMP phosphodiester intermediate (Fig. 2a,b)^3^. Other adenylate forming enzymes, including non-ribosomal peptide synthetases, acyl-CoA ligases, luciferases, and tRNA synthetases, have been targeted by small molecule inhibitors that mimic the acyl-AMP phosphodiester intermediate. These inhibitors replace the native phosphate moiety with a non-hydrolysable sulfamoyl moiety, which is resistant to further enzymatic reactions^23^. They also include a mimetic substrate specific to the enzyme being targeted, shown in blue in Fig. 2. These molecules bind tightly to the enzyme active site as intermediate mimics but are not susceptible to nucleophilic attack by an incoming hydroxylate or thiolate. AasS enzymes have been shown to efficiently load short and medium chain fatty acid (C4–C12) substrates with a chain length preference of C10, and less efficiently long chain fatty acids (C14–C18)^27,28^. We therefore chose to design our inhibitor, C10-AMS, to mimic a medium chain fatty acid with three key regions: adenosine, C10 acyl chain, and a non-hydrolysable sulfamoyl linker (Fig. 2c).

**Figure 3:**
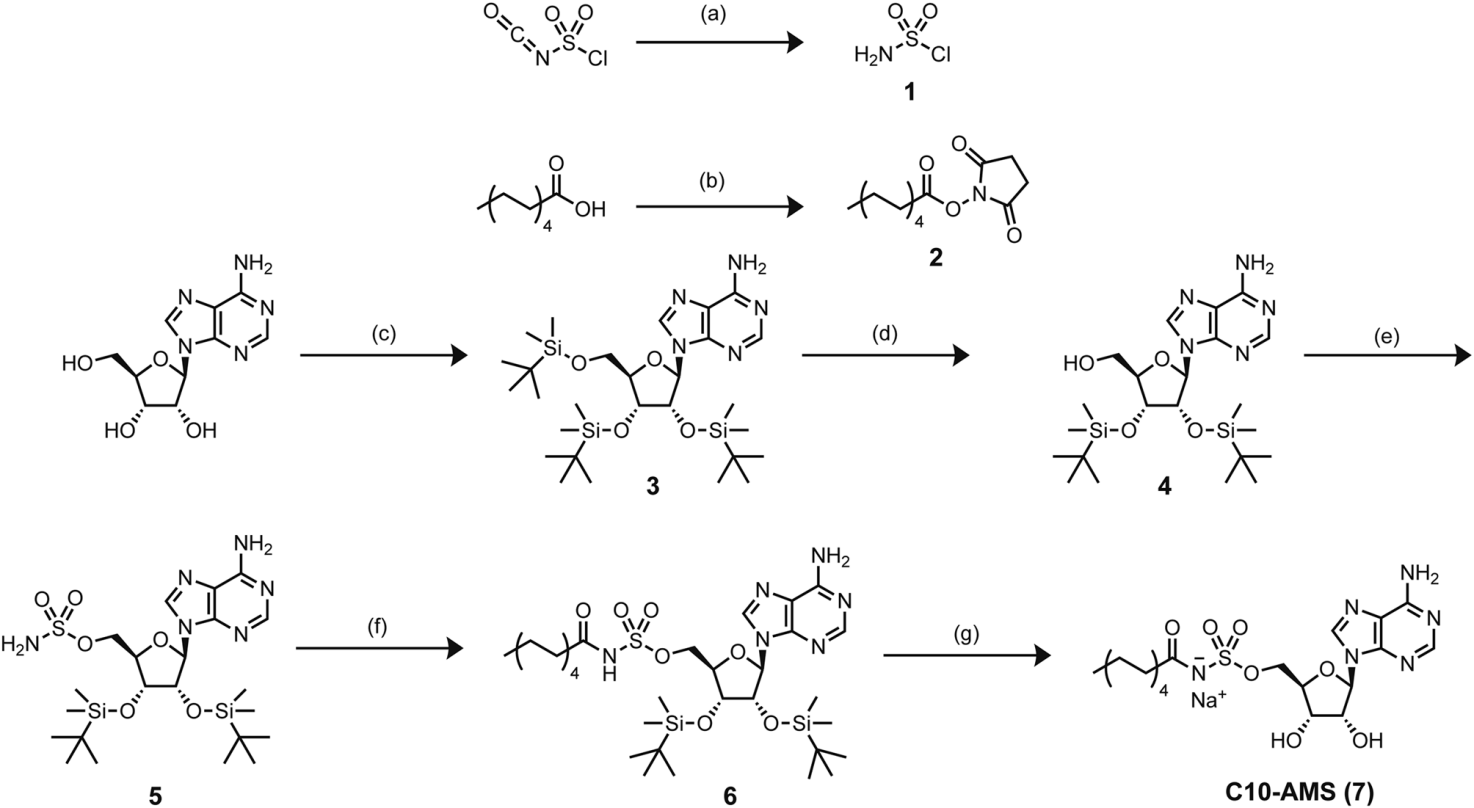
Synthetic scheme of C10-AMS (7) inhibitor. (**a**) HCO_2_H, DCM; (**b**) NHS, EDC, DCM; (**c**) imidazole, TBSCl, DMF; (**d**) TFA, THF, H_2_O; (**e**) **1**, DMA; (**f**) **2**, DBU, DMF; (**g**) HCl, dioxane, H_2_O then NaOH, MeOH.

The synthetic scheme used to prepare C10-AMS in milligram quantities, outlined in Fig. 3 and the methods section, was based generally on published procedures^20–22,29^. Commercially available adenosine was converted into 2′,3′-*O,O*-Bis(*t*-butyldimethylsilyl)adenosine (**4**) over two steps^20,21^, and then coupled with sulfamoyl chloride (**1**) to yield 2′,3′-*O,O*-Bis(*t*-butyldimethylsilyl)-5′-*O*-sulfamoyladenosine (**5**)^20,22^. The acyl chain was then incorporated using the activated N-hydroxysuccinimide (NHS) ester of decanoic acid. Final deprotection of the silyl groups was achieved with HCl in dioxane^20^, which we found to have an improved yield over the alternative conditions using TBAF^21,29^, to produce the inhibitor C10-AMS. A key step in the synthetic pathway was the initial formation of the sulfamoyl chloride (**1**), which is both an extremely exothermic and air sensitive reaction. Ensuring the reaction mixture and product never come into contact with air is essential to the successful formation of the product and subsequent coupling with **4**. Purification and characterization of C10-AMS was challenging as the introduction of the C10 acyl chain was far more nonpolar than other inhibitor substrates^23^. Purification was done by manipulating C10-AMS’s solubility in various solvents. C10-AMS was also converted into its conjugate base for stability, since the negative charge on the nitrogen deters intramolecular nucleophilic attack on the carbonyl moiety^20,22^. All intermediates and final products were characterized by ^1^H NMR and LCMS (Supporting Information).

### Expression and purification of ACP and AasS

For biochemical studies, two ACPs and two AasSs were chosen—one as a well-studied model system and the other as an example system from a gram-negative pathogenic organism. *Escherichia coli* has long been a model organism to study bacterial pathways, and its ACP (EcACP) was used for these experiments. *E. coli* does have an atypical AasS that is bifunctional, however, it is both unstable in vitro and not able to recycle exogenously supplied fatty acids^16,30–32^. The first described soluble AasS is from the gram-negative marine bacterium *Vibrio harveyi* (VhAasS). This enzyme is the most thoroughly studied in this class and a good model for this study^17,27,28^. VhAasS is promiscuous for its ACP partner, and it can load a broad array of acyl chains onto EcACP. The acyl chain length preference for VhAasS is 8 to 14 carbons. When overexpressed in *E*. *coli* it incorporates exogenous fatty acids into FAS, which get elongated and incorporated into lipids^28^. Our group recently discovered another AasS from the gram-negative bacterium *Vibrio cholerae* (VcAasS), the causative agent of the disease cholera (unpublished data). This AasS and the cognate ACP (VcACP) from *Vibrio cholerae* were chosen as a pair from a pathogenic organism.

When ACP is expressed as a His_6_-tagged construct in *E. coli* BL21[DE3], a mixture of *apo*- and *holo*-ACP is obtained. *Apo*-ACP is unmodified, while *holo*-ACP has been post-translationally modified with the 4′-phosphopantetheine moiety from CoA by a phosphopantetheinyl transferase. *E. coli* has a native phosphopantetheinyl transferase, AcpS, that is able to partially convert *apo*-ACP to *holo*-ACP during over-expression of ACP. Since AasS uses the terminal thiol on the 4′-phosphopantetheine arm of ACP, it is essential for ACP to be in its *holo* form. Therefore, the mixture for both EcACP and VcACP was purified by Ni–NTA affinity chromatography and subsequently converted to *holo*-ACP by a promiscuous phosphopantetheinyl transferase, Sfp, from *Bacillus subtilis*^33^. VhAasS and VcAasS were also expressed as a His_6_-tagged construct in *E. coli* BL21[DE3] and purified by Ni–NTA affinity chromatography.

### In vitro inhibition of AasS loading of fatty acids

We tested the ability of C10-AMS to inhibit the AasS-catalyzed loading of fatty acids onto ACP in vitro. Loading was monitored by conformationally sensitive urea-PAGE gel and mass spectrometry. ACPs are small proteins, approximately 10 kDa, that have a characteristic four alpha helical bundle, and they sequester acyl chains attached to them for protection from hydrolysis and unwanted reactions. As ACP tightens around the attached acyl chain, it has an apparent smaller size and migrates further on urea-PAGE. Therefore, *holo*-ACP (not loaded with an acyl chain by AasS) and acyl-ACP (loaded with an acyl chain by AasS) can be distinguished by different gel shifts^34^. In addition, LCMS has been used to distinguish *holo*- and acyl-ACPs^28^. Four combinations of proteins were tested, each with decanoic acid (C10) as a substrate: (1) EcACP and VhAasS, (2) EcACP and VcAasS, (3) VcACP and VhAasS, and (4) VcACP and VcAasS.

**Figure 4:**
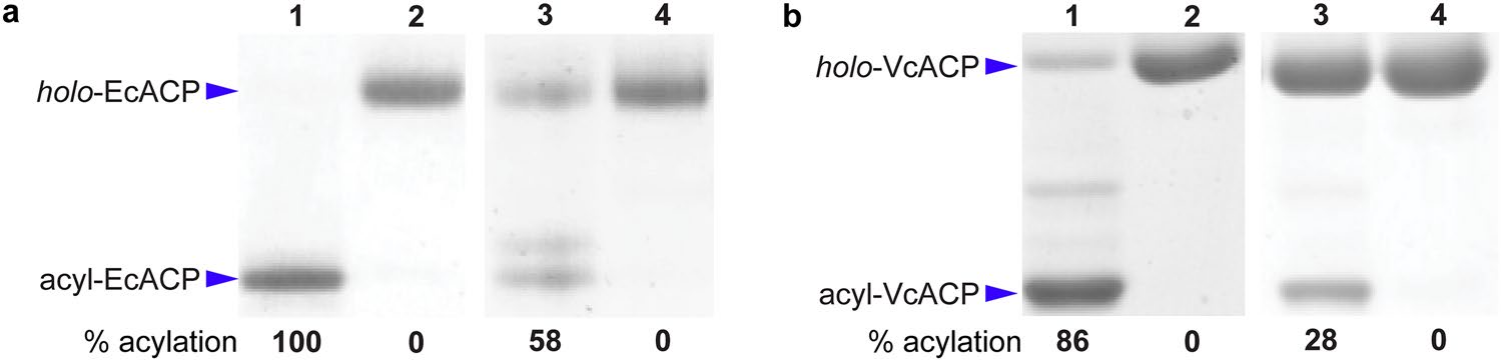
In vitro AasS loading onto ACP and inhibition with C10-AMS. (**a**) Urea-PAGE loading of decanoic acid onto EcACP with VhAasS (lane 1) and with VcAasS (lane 3) and inhibition with C10-AMS (lanes 2 and 4). (**b**) Urea-PAGE loading of decanoic acid onto VcACP with VhAasS (lane 1) and with VcAasS (lane 3) and inhibition with C10-AMS (lanes 2 and 4). Percent acylation (%) for each reaction was determined by densitometry using ImageJ and is reported under each lane. Final concentrations: EcACP and VcACP (30 µM), VhAasS (0.5 µM), VcAasS (30 µM), decanoic acid and C10-AMS (1 mM). Full-length gels are presented in Supplementary Fig. S1.

VhAasS has been shown in the literature to be highly active and promiscuous^28^, and our results show it is able to load decanoic acid onto both EcACP (100% )and VcACP (86%) in good yield as seen in lane 1 and determined by densitometry using Image J (Fig. 4a,b)^35^. In the presence of C10-AMS, VhAasS is completely inhibited from loading decanoic acid onto both EcACP and VcACP as seen in lane 2 (Fig. 4a,b). VcAasS is less active, and even at much higher concentrations the enzyme is not able to achieve 100% loading of fatty acids onto ACP. However, VcAasS was able to load decanoic acid onto EcACP (58%) and VcACP (28%) as seen in lane 3 and determined by densitometry using ImageJ (Fig. 4a,b)^35^. When loading decanoic acid in the presence of C10-AMS, VcAasS is completely inhibited from loading its fatty acid substrate onto both EcACP and VcACP, as seen in lane 4 (Fig. 4a,b). Extra bands in lane 3 (Fig. 4a) and lane 1 (Fig. 4b) are also acyl-ACP, likely loaded with a different chain length fatty acid found in trace amounts in the reaction conditions, and the formation of these species are additionally inhibited by C10-AMS as seen in lanes 2 and 4.

### In vivo studies of AasS’s role in fatty acid recycling

**Figure 5:**
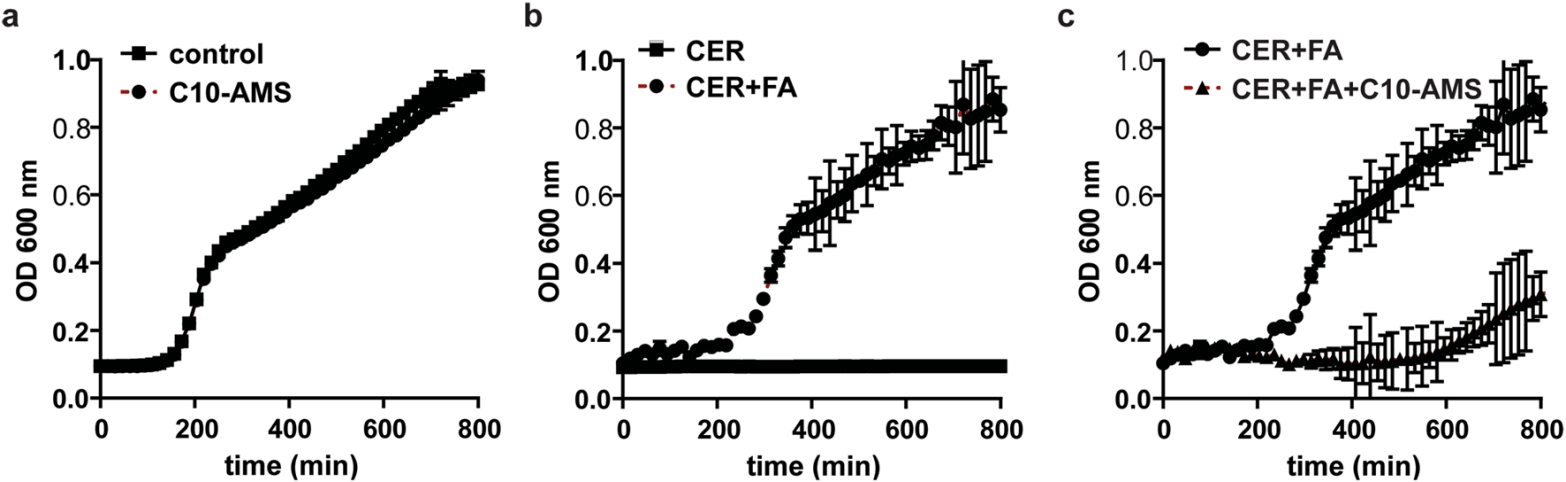
Growth curves of *V. cholerae* in the presence of cerulenin, fatty acids and C10-AMS. (**a**) C10-AMS at a concentration of 50 µM does not inhibit growth of *V. cholerae*. (**b**) Cerulenin at a concentration of 5 µM inhibits growth but addition of 1 mM of a mixture of fatty acids rescues growth. (**c**) Addition of 50 µM C10-AMS to *V. cholerae* treated with 5 µM cerulenin and 1 mM of a mixture of fatty acids inhibits growth.

Since C10-AMS effectively inhibited AasS from loading fatty acids onto ACP in vitro, we wondered whether C10-AMS could be utilized as an antibiotic. To test this, *V. cholerae* was used as a model pathogenic organism. In rich media, C10-AMS at a concentration of 50 µM has no effect on growth (Fig. 5a). Under those conditions, bacteria produce fatty acids via route 1, endogenous production by FAS, and therefore the recycling of exogenous fatty acids is not required. As predicted an inhibitor of AasS, which recycles fatty acids, has no effect. FAS must be inhibited to make it necessary for the bacteria to utilize exogenous fatty acids. Next we used cerulenin, a potent inhibitor of a key FAS enzyme, at a concentration of 5 µM to inhibit FAS and kill *V. cholerae* (Fig. 5b). Because endogenous production of fatty acids is inhibited and there are no fatty acids supplied in the media to recycle, *V. cholerae* is unable to build its cell membranes and survive. We have shown recently that supplying exogenous fatty acids can rescue treatment of *V. cholerae* with several FAS-targeted antibiotics. Here, a mixture of short and long chain fatty acids (1 mM) rescues inhibition by cerulenin (Fig. 5b). Finally, we added C10-AMS at a concentration of 50 µM to a culture treated with cerulenin and fatty acids and inhibition of growth was observed (Fig. 5c), suggesting that when bacteria have their endogenous production of fatty acids by FAS inhibited, and are therefore required to uptake and utilize exogenous fatty acids, C10-AMS functions as an antibiotic.

## Discussion

Fatty acid biosynthesis by FAS in bacteria has long been an antibiotic target, and potent inhibitors like triclosan are currently in use, while others are in clinical development^8–13^. However, some bacteria are able to bypass inhibition by recycling fatty acids from their environment (without the need to break them down) to use in lipid cell membranes, allowing them to survive. AasS efficiently catalyzes the activation of exogenous fatty acids and subsequent placement onto *holo*-ACP, a critical step for fatty acid incorporation into lipids^3,6,7^. While other adenylate forming enzymes have been targets of inhibitors, we found only a couple of papers attempting to target an AasS type enzyme^29,36^. Interestingly, these papers are targeting enzymes in *Mycobacterium tuberculosis* denoted as fatty acyl AMP ligases (FAAL). In *M. tuberculosis*, these enzymes activate fatty acids by converting them into AMP ester intermediates, but then load them onto ACP from polyketide synthases (PKS) rather than FAS. FAALs therefore catalyze the same two reactions as AasS, but are functionally unique to polyketide biosynthesis and not used for fatty acid recycling^36^. Only five AasSs have been characterized to date, but based on bioinformatics the presence of this enzyme in bacteria might be widespread^28^. In general, AasSs have striking similarity to long chain fatty acid acyl-CoA ligases, which also convert fatty acids into an activated AMP ester intermediate, but subsequently load it onto the small molecule CoA rather than ACP. Because these enzymes are so similar, they cannot be distinguished by sequence alone. We hypothesize that many AasS enzymes are annotated as fatty acyl AMP ligases, acyl-CoA ligases, and FadD homologous enzymes^28^. AasS is not essential to bacteria and thus not generally considered a good antibiotic target. However, there is interest in compounds that inhibit non-essential bacterial targets, including virulence factors, since bacteria seem to develop resistance to these drugs at lower rates^37^. The role of AasS in fatty acid recycling should be further explored as an antibiotic target in conjunction with FAS inhibitors. As we continue to learn more about the AasS enzyme and discover it in a range of organisms, having a tool to inhibit its activity and study it in in vitro and in vivo is valuable.

Here we show that C10-AMS is able to effectively stop in vitro loading of decanoic acid onto ACPs from *E. coli* and *V. cholerae* by inhibiting AasS from both *V. harveyi* and *V. cholerae*, even though they have differing activity (Fig. 4). C10-AMS is designed to mimic one of the substrates of AasS, but with a non-hydrolysable sulfamoyl moiety instead of a hydrolysable phosphate moiety, and acts as a competitive inhibitor (Fig. 2c). C10-AMS was used at a concentration that matched the fatty acid substrate, and pre-incubation with AasS was not required to see complete inhibition. We should note that during the review of this manuscript, Baran et al. also synthesized C10-AMS via a different route, and it was found to have good inhibition of FAALs from *Mycobacterium tuberculosis* as well^36^. We are excited to see that inhibition translates from in vitro to in vivo. Since bacteria produce their own fatty acids via route 1, endogenous production by FAS, recycling fatty acids is not essential to their survival, as seen by C10-AMS having no effect on the growth of *V. cholerae.* However, when FAS is targeted with known inhibitors like cerulenin, bacteria are unable to survive unless they are in an environment with a range of fatty acids that they can recycle and incorporate into lipids. When in a fatty acid rich environment, but also fed cerulenin and C10-AMS, *V. cholerae* is unable to survive (Fig. 5). In this case, both de novo fatty acid production and the ability to recycle fatty acids are stopped, and without this essential membrane building block the pathogenic bacteria cannot persist. IC50 values with related sulfamoyl adenosine inhibitors have ranged from nM to mM^21,25,38–40^, therefore inhibition with 50 µM is within range and suggests a competitive, non-covalent mechanism of inhibition. C10-AMS is not necessarily specific to VcAasS, as it may target acyl-CoA ligases or even other undiscovered AasS enzymes in *V. cholerae*. Perhaps the modest recovery of growth after 10 h in the presence of FAS inhibitor, fatty acids, and C10-AMS (Fig. 5c) might be due to overexpression of one or several of these enzymes. Regardless, this inhibitor does stop the ability to recycle fatty acids, so we can conclude that these adenylate forming enzymes play an active role in this process. Even as a non-selective antibiotic, C10-AMS allows us to test AasS inhibition in vitro and the role of this enzyme class in other pathogenic organisms to further study fatty acid recycling and develop potential antibiotics.

## Methods

### General synthetic methods

All commercial reagents were obtained from Sigma-Aldrich, Fisher or Thomas Scientific. All reactions were carried out under inert atmosphere in dry solvents with oven-dried glassware and constant magnetic stirring unless otherwise noted. TLC analysis was performed using Silica Gel 60 F254 plates 9EM Scientific and visualization was accomplished using UV light. Silica gel chromatography was carried out with Silicycle 60 230–400 mesh or using a Biotage flash purification system. ^1^H-NMR spectra were taken on a JEOL 400 ECS and data was processed with Delta V5.

### Synthesis of C10-AMS inhibitor

The synthetic scheme can be seen in Fig. 3 and characterization can be found in the Supporting Information.

#### Sulfamoyl chloride (1)

Chlorosulfonyl isocyanate (4 mL, 46 mmol) in 12 mL dry dichloromethane (DCM) was added to a 250 mL two-necked round bottom flask equipped with a drying tube and put under nitrogen. The flask was cooled to 0 °C and a solution of formic acid (1.7 mL, 46 mmol) in 9.5 mL dry DCM was added dropwise over 10 min. The reaction mixture was warmed to room temperature and stirred overnight. The DCM was removed under nitrogen with a syringe, the white solid was washed with precooled (at − 80 °C) dry DCM, and the solvent was again removed under nitrogen.

#### Succinimidyl decanoate (2)

Decanoic acid (1.00 g, 5.8 mmol) and N-hydroxysuccinimide (668 mg, 5.8 mmol) in 15 mL DCM and 0.1 mL dimethyl formamide (DMF) were added to a 100 mL round bottom flask. 1-Ethyl-3-(3-dimethylaminopropyl)carbodiimide (1.11 g, 5.8 mmol) was added and the reaction mixture was stirred at 0 °C for 3 h and 4 °C overnight. The reaction mixture was washed with 30 mL water and extracted with 3 × 30 mL DCM, dried over Na_2_SO_4_, concentrated by rotary evaporation, and recrystallized from ethanol (753 mg, 48% yield).

#### 2′,3′,5′-*O,O,O*-tris(*t*-butyldimethylsilyl)adenosine (3)

(-)-Adenosine (1.00 g, 3.7 mmol) and imidazole (2.29 g, 33 mmol) were added to a 50 mL round bottom flask, put under nitrogen, and 5 mL dry DMF was added. tert-butyldimethylsilyl chloride (1.97 g, 13 mmol) was dissolved in 3 mL dry DMF and added to the stirring solution dropwise over 3 min at room temperature. The reaction mixture was stirred for 30 h and diluted with 50 mL DCM. The reaction mixture was washed with 3 × 50 mL saturated sodium bicarbonate, dried over Na_2_SO_4_, and concentrated by rotary evaporation (1.99 g, 87% yield).

#### 2′,3′-*O,O*-Bis(*t*-butyldimethylsilyl)adenosine (4)

**3** (0.71 g, 1.2 mmol) was added to a 50 mL pointed flask in 14 mL tetrahydrofuran and cooled to 0 °C. A solution of trifluoroacetic acid (3 mL) in water (3 mL) was added and the reaction mixture was stirred for 5 h at 0 °C. The mixture was diluted with 30 mL saturated sodium bicarbonate at 0 °C then extracted with 3 × 40 mL ethyl acetate (EtOAc). The organic layer was washed with 30 mL brine, dried over Na_2_SO_4_, and concentrated by rotary evaporation. The product was purified by flash chromatography; loaded in DCM and eluted with the following gradient: 300 mL 1:1 EtOAc/Hexanes, 300 mL 3:1 EtOAc/Hexanes (458 mg, 79% yield).

#### 2′,3′-*O,O*-Bis(*t*-butyldimethylsilyl)-5′-*O*-sulfamoyladenosine (5)

**4** (0.40 g, 0.8 mmol) was added to a 20 mL pointed flask, put under nitrogen, and 4 mL dry dimethylacetamide was added and cooled to 0 °C. Sulfamoyl chloride (375 mg) was added dry and the reaction mixture was stirred at 0 °C for 1 h and room temperature for 4 h. The reaction mixture was diluted with 25 mL EtOAc and 15 mL water. The aqueous layer was neutralized with solid sodium bicarbonate to pH 7 and extracted with 3 × 25 mL EtOAc. The organic layer was dried over Na_2_SO_4_ and concentrated by rotary evaporation. The product was purified by flash chromatography; loaded in DCM and eluted with the following gradient: 200 mL 15:1 DCM/methanol, 200 mL 9:1 DCM/methanol (402 mg, 86% yield).

#### 2′,3′-*O,O*-Bis(*t*-butyldimethylsilyl)-5′-*O*-(*N*-decanylsulfamoyl)adenosine (6)

**2** (56 mg, 0.2 mmol) was added to a 20 mL pointed flask and put under nitrogen. **5** (100 mg, 0.17 mmol) was dissolved in 2 mL dry DMF and added to the flask. The reaction mixture was cooled to 0 °C and 1,8-diazabicylco(5.4.0)undec-7-ene (DBU) (31 μL, 0.21 mmol) was added. The reaction mixture was allowed to warm to room temperature and stirred overnight. The reaction mixture was evaporated and dissolved in 15 mL water, then extracted with 3 × 30 mL EtOAc. The organic layer was washed with 20 mL brine, dried over Na_2_SO_4_, and concentrated by rotary evaporation. The product was purified by flash chromatography; loaded in 9:1 DCM/methanol and eluted with 150 mL 9:1 DCM/methanol (105 mg, 83% yield).

#### 5′-*O*-(*N*-decanylsulfamoyl)adenosine mono sodium salt (7, C10-AMS)

**6** (145 mg, 0.20 mmol) was added to a 20 mL pointed round bottom flask, put under nitrogen and 0.7 mL dioxane and 0.5 mL water were added. The flask was cooled to 0 °C and 1.1 mL 2.5 M HCl in dioxane was added dropwise. The reaction mixture was stirred for 5 min at 0 °C and room temperature for 8 h. The reaction mixture was evaporated. 3 × 5 mL methanol was added and evaporated. 1.5 mL methanol and 1.0 mL 1 M sodium hydroxide was added to basify the solution to pH 9 and evaporated. The product was purified by dissolving in 9:1 MeOH/DCM and filtering out yellow byproducts (20 mg, 20% yield).

### Protein expression and purification

*Escherichia coli* ACP (EcACP), *Vibrio cholerae* ACP (VcACP), *Vibrio harveyi* AasS (VhAasS), *Vibrio cholerae* AasS (VcAasS), and *Bacillus subtilus* Sfp were expressed as His_6_-tagged constructs in *E. coli* BL21[DE3], 1 L cultures in Luria broth media (LB) with 100 mg/mL ampicillin or 50 mg/mL kanamycin were grown at 37 °C to an optical density at 600 nm (OD600) of 0.8, cooled to 16 °C, induced by the addition of isopropyl β-d-1-thiogalactopyranoside (IPTG) to 0.1 mM, and shaken for 18 h. Cells were harvested via centrifugation. The cell pellet was re-suspended in 30 mL buffer A (50 mM phosphate pH 7.5, 150 mM NaCl, 10% glycerol) with 1 mg/mL lysozyme, nutated at 4 °C for 1 h, and sonicated. The lysate was cleared via centrifugation at 15,000 × g for 30 min. The cleared lysate was batch-bound to 2 mL Ni resin and incubated 1 h at 4 °C. The resin was washed with 100 mL buffer A followed by 100 mL buffer A containing 25 mM imidazole. The protein was eluted with buffer A containing 250 mM imidazole, dialyzed overnight into buffer A, concentrated, and stored at − 80 °C.

### Acyl carrier protein conversions and inhibition

A mixture of *apo*/*holo*-ACP (EcACP and VcACP, 69 µM) was transformed into pure *holo*-ACP by incubation with Sfp (1.2 µM), MgCl_2_ (12.5 mM), TCEP (5 mM), and CoA (2 mM) in Buffer A for 18 h at 37 °C. Conversion was confirmed by urea-PAGE. Loading of fatty acids onto *holo*-ACP to produce acyl-ACP by AasS was performed with EcACP or VcACP (30 µM), decanoic acid (1 mM), MgCl_2_ (12.5 mM), ATP (10 mM), TCEP (2.5 mM), and VhAasS (0.5 µM) or VcAasS (30 µM) in Buffer A for 24 h at 37 °C. Inhibition was tested under the same conditions with the addition of C10-AMS (1 mM).

### Acyl carrier protein urea-PAGE and liquid chromatography mass spectrometry

We relied on conformationally sensitive urea-PAGE analysis to distinguish *apo*-, *holo*-, and acyl-ACP formation^34^. Samples were mixed with bromophenol blue load dye excluding SDS, loaded onto the gel, and run for 2 h at 180 V. Protein bands were visualized with Bio-Safe Coomassie stain.

ACPs were additionally analyzed on a Waters Acquity I-Class UPLC system coupled to a Synapt G2Si HDMS QTOF mass spectrometer in positive ion mode with a heated electrospray ionization source. LC separation was performed on a Waters Acquity UPLC BEH 1.7 µm 2.1 × 50 mm column using an 0.6 ml/min gradient of 95/5 to 15/85 A/B in 4 min. Eluent A is 0.1% formic acid in water and B is 0.1% formic acid in ACN. Low and high energy masses were collected and the ESI mass spectra deconvoluted using Maxent1. For phosphopantetheine ejection we analyzed the high energy data using Masslynx^41^.

### Growth curves of *V. cholera*

All growth curves were performed in 96-well plates shaking at 37 °C using a microplate reader (Tecan) with absorbance measured at 600 nm. A total of 200 µl of LB medium with and without supplements were aliquoted per well. Overnight *V. cholerae* cultures grown in LB were inoculated at a 1:1000 dilution. The mixture of fatty acids contains short chain and long chain fatty acids (C3, C4, C14, C16, C18, C18:1). All experiments were performed in biological duplicate and repeated twice.

## Supplementary information

Supplementary Information.
