# Supplementary material for: Synthesis of an acyl-acyl carrier protein synthetase inhibitor to study fatty acid recycling
Source: Sci Rep. 2020 Oct 20;10:17776. doi: 10.1038/s41598-020-74731-4 (PMC7575536; doi:10.1038/s41598-020-74731-4)

Synthesis of an acyl-acyl carrier protein synthetase inhibitor to study fatty acid recycling

Madeline F. Currie[1], Dylan M. Persaud[1], Niralee K. Rana[1], Amanda J. Platt[2], Joris Beld[2*], and Kara L. Jaremko[1*]

[1]Department of Chemistry
Hofstra University
Hempstead, New York 11549 (USA)
Kara.L.Jaremko@hofstra.edu

[2]Department of Microbiology and Immunology
Drexel University College of Medicine
Philadelphia, PA 19102 (USA)
jb3669@drexel.edu

Supporting Information Contents

Figure S1. Uncropped gel images of *in vitro* AasS loading onto ACP and inhibition with C10-AMS, corresponding to main text Figure 4 2

Figure S2. Liquid chromatography mass spectrometry (LCMS) analysis of loading of decanoic acid onto ACP with AasS and inhibition with C10-AMS 3

Compound Characterization and ^{1}H NMR Spectra 4-6

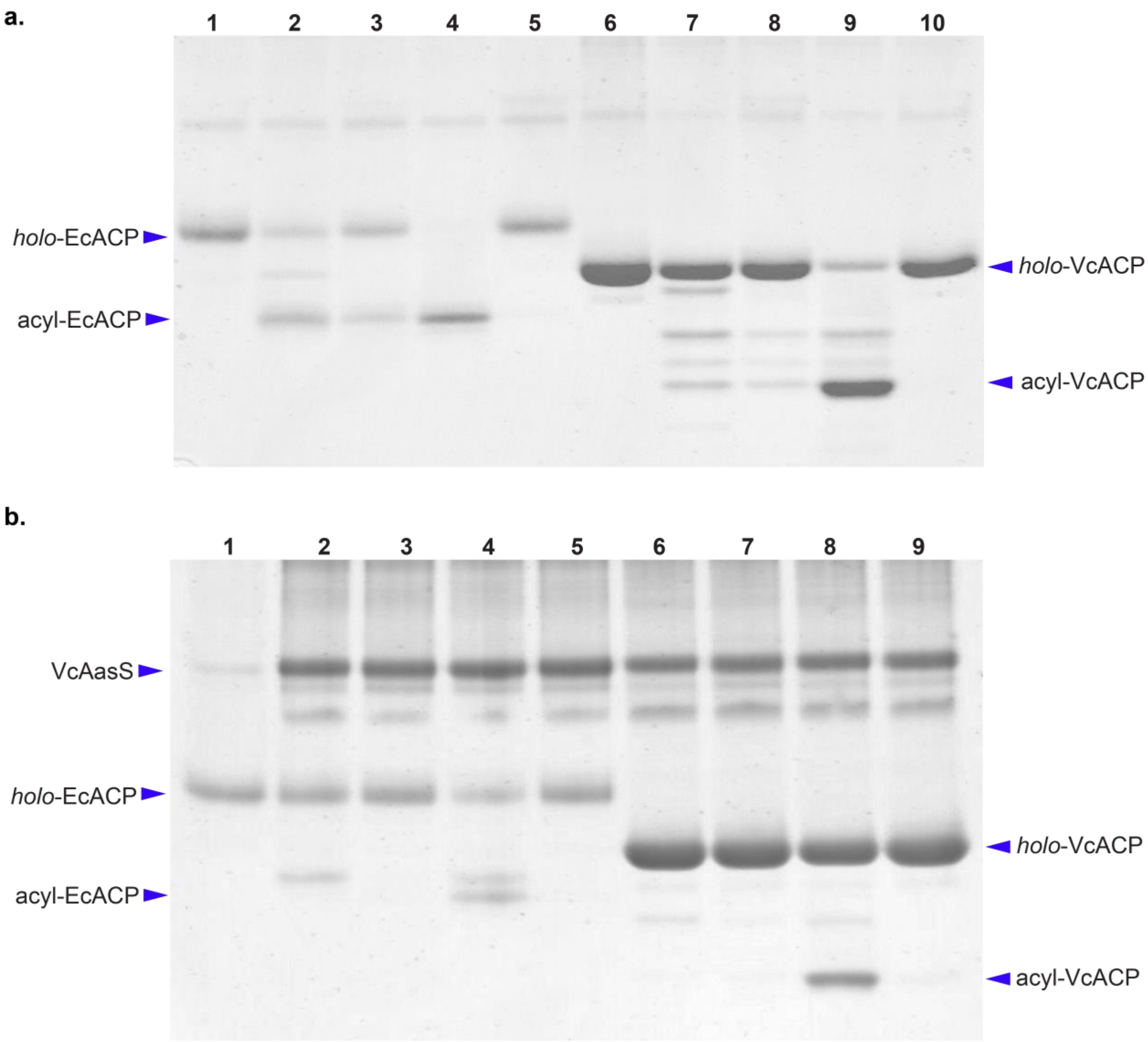

Figure S1. Uncropped gel images of *in vitro* AasS loading onto ACP and inhibition with C10-AMS, corresponding to main text Figure 4. (a) Urea-PAGE loading of decanoic acid with VhAasS onto EcACP (lane 4) and VcACP (lane 9) and inhibition with C10-AMS (lanes 5 and 10). (b) Urea-PAGE loading of decanoic acid with VcAasS onto EcACP (lane 4) and VcACP (lane 8) and inhibition with C10-AMS (lanes 5 and 9). In panel (a), lanes 1 and 6 are EcACP and VcACP standards, respectively. Lanes 2,3,7, and 8 represent loading of butyric acid and inhibition, results not included in this manuscript. In panel (b), lane 1 is EcACP standard. Lanes 2,3,6, and 7 represent loading of butyric acid and inhibition, results not included in this manuscript.

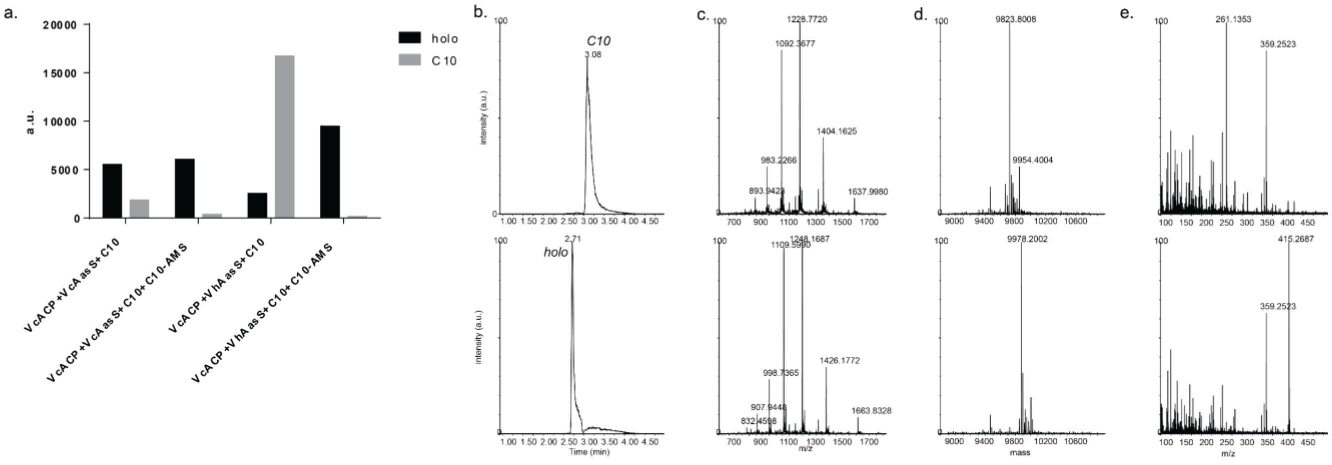

Figure S2. Liquid chromatography mass spectrometry (LCMS) analysis of loading of decanoic acid onto ACP with AasS and inhibition with C10-AMS. (a) Peak integration of *holo*-VcACP and C10 acyl-VcACP upon loading with either VcAasS or VhAasS and decanoic acid in the presence and absence of C10-AMS. (b) Extracted ion chromatogram of *holo*-VcACP (top) and C10 acyl-VcACP (bottom). (c) High resolution mass spectra of *holo*-VcACP (top) and C10 acyl-VcACP (bottom). (d) Deconvoluted ESI mass spectra of *holo*-VcACP (top) and C10 acyl-VcACP (bottom). (e) Zoom-in on low molecular masses for *holo*-VcACP (top) and C10 acyl-VcACP (bottom) to visualize phosphopantetheine ejections peaks for *holo* (359.2523 and 261.1353) and C10 acyl (415.2687 and 359.2687).

Succinimidyl decanoate (2): ^{1}H-NMR ($CDCl_3$, 400MHz) $\delta = 2.81$ (d, 4H), 2.57 (t, 2H), 1.71 (m, 2H), 1.18-1.42 (m, 12H), 0.85 (t, 3H).

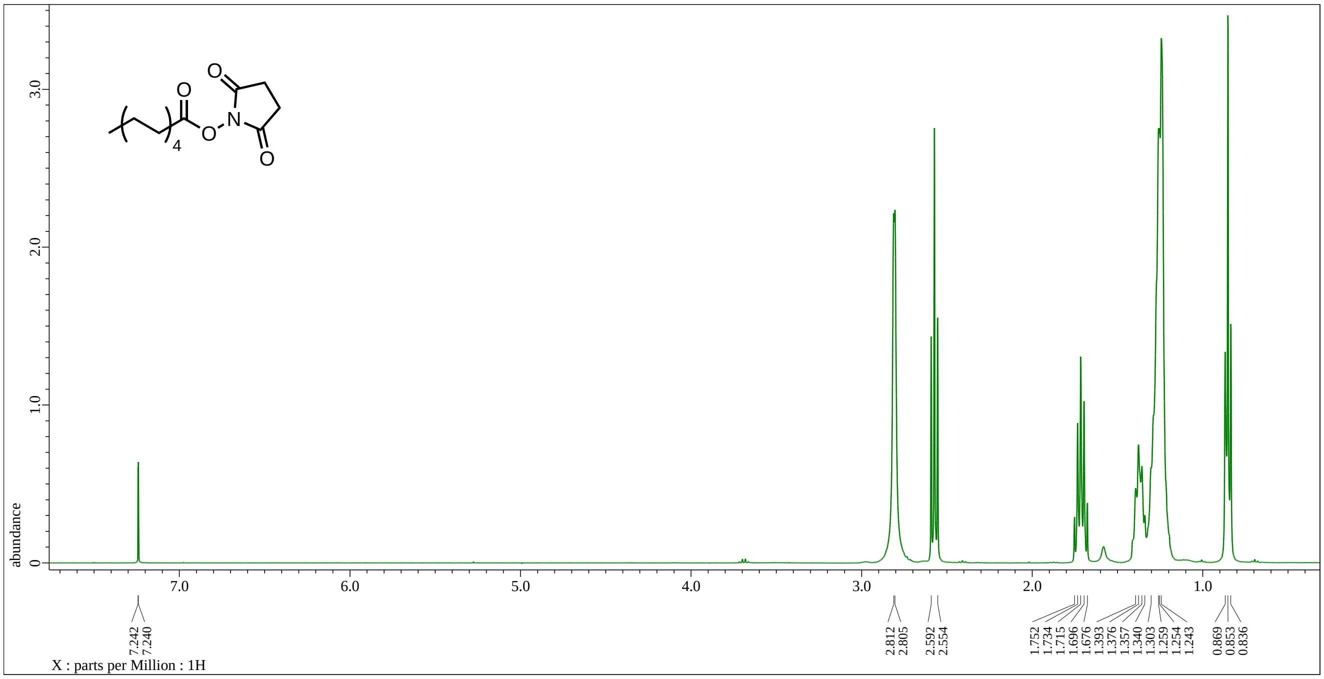

2',3',5'-*O,O,O*-tris(*t*-butyldimethylsilyl)adenosine (3): ^{1}H-NMR ($CDCl_3$, 400MHz) $\delta = 8.24$ (s, 1H), 8.08 (s, 1H), 5.95 (d, 1H), 5.68-5.77 (s, 1H), 4.55 (t, 1H), 4.20 (t, 1H), 4.02 (q, 1H), 3.93 (dd, 1H), 3.68 (dd, 1H), 0.85 (s, 9H), 0.83 (s, 9H), 0.69 (s, 9H), 0.04 (s, 3H), 0.03 (s, 3H), 0.00 (s, 3H), -0.01 (s, 3H), -0.14 (s, 3H), -0.32 (s, 3H). ESI-MS m/z: (pos) 610.36 $[M+H]^+$

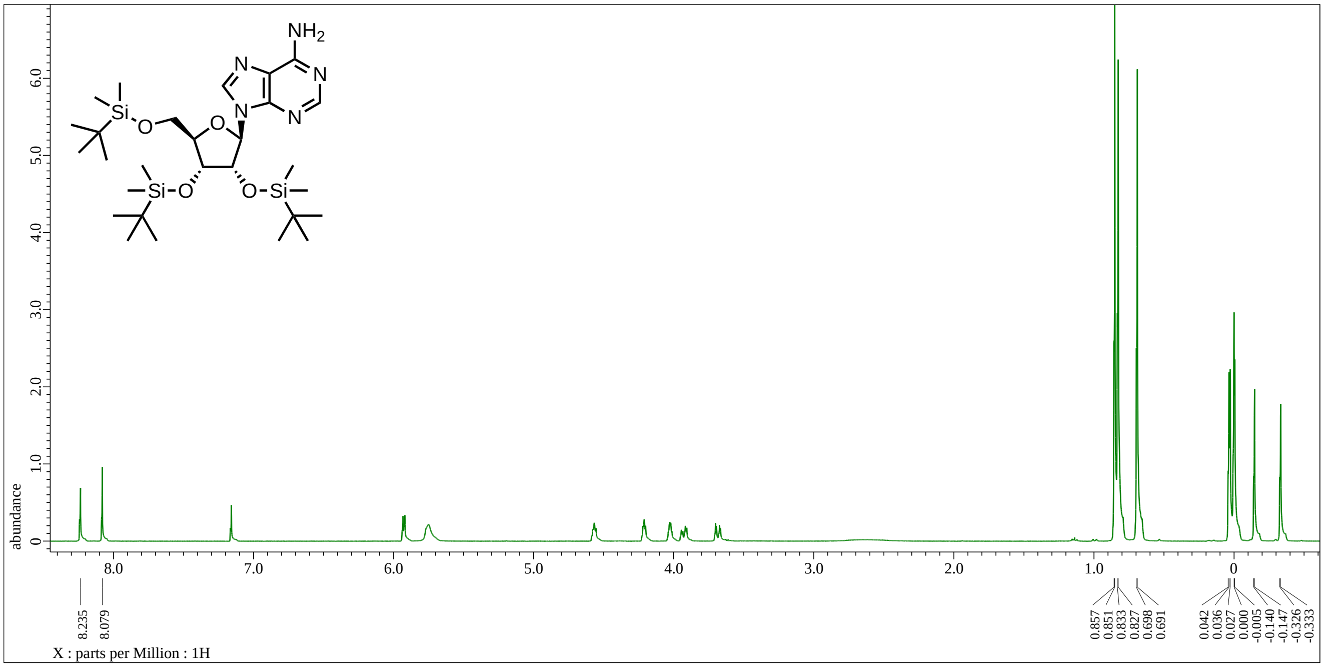

2',3'-*O,O*-Bis(*t*-butyldimethylsilyl)adenosine (4)*:* ^{1}H-NMR ($CDCl_3$, 400MHz) δ = 8.22 (s, 1H), 7.77 (s, 1H), 6.05-6.15 (s, 1H), 5.70 (d, 1H), 4.90 (q, 1H), 4.22 (d, 1H), 4.05 (s, 1H), 3.83 (d, 1H), 3.58 (d, 1H), 0.83 (s, 9H), 0.63 (s, 9H), 0.01 (s, 3H), 0.00 (s, 3H), -0.24 (s, 3H), -0.71 (s, 3H). ESI-MS m/z: (pos) 496.28 [M+H]$^+$

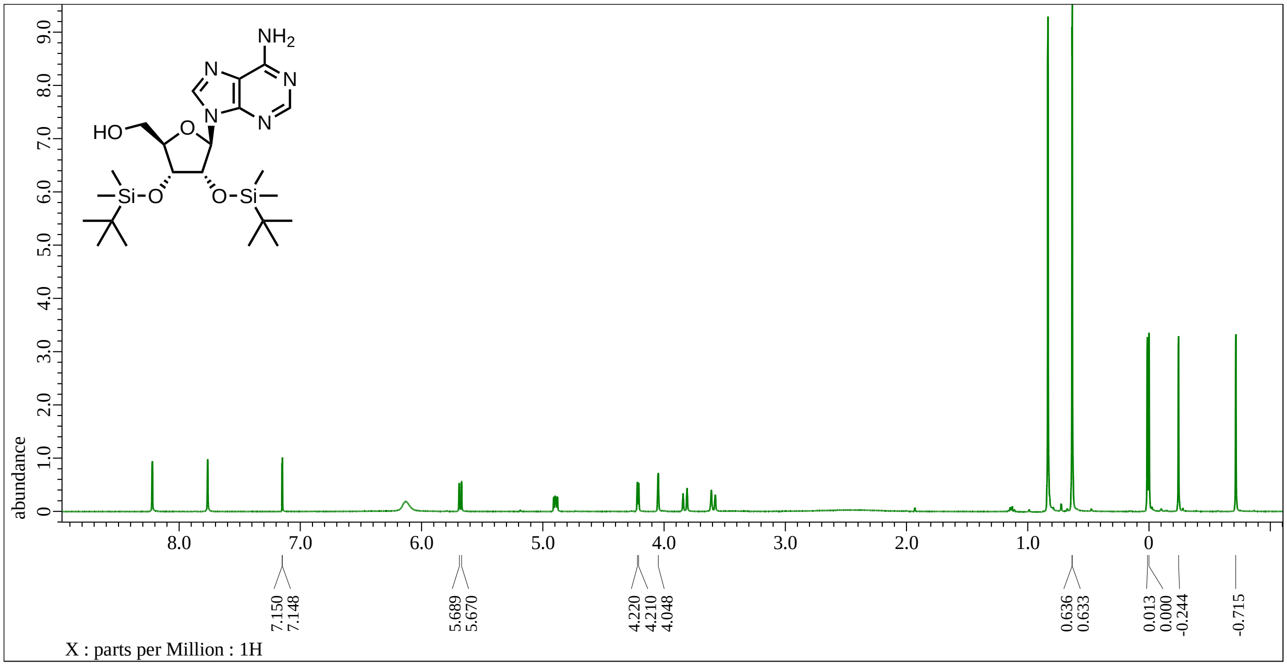

2',3'-*O,O*-Bis(*t*-butyldimethylsilyl)-5'-*O*-sulfamoyladenosine (5): ^{1}H-NMR (DMSOd$_6$, 400MHz) δ =8.44 (s, 1H), 8.24 (s, 1H), 7.73 (s, 2H), 7.41 (s, 2H), 6.03 (d, 1H), 5.05 (q, 1H), 4.45-4.51 (m, 2H), 4.37-4.42 (q, 1H), 4.26 (t, 1H), 1.01 (s, 9H), 0.79 (s, 9H), 0.23 (s, 3H), 0.21 (s, 3H), 0.00 (s, 3H), -0.29 (s, 3H). ESI-MS m/z: (pos) 575.25 [M+H]$^+$

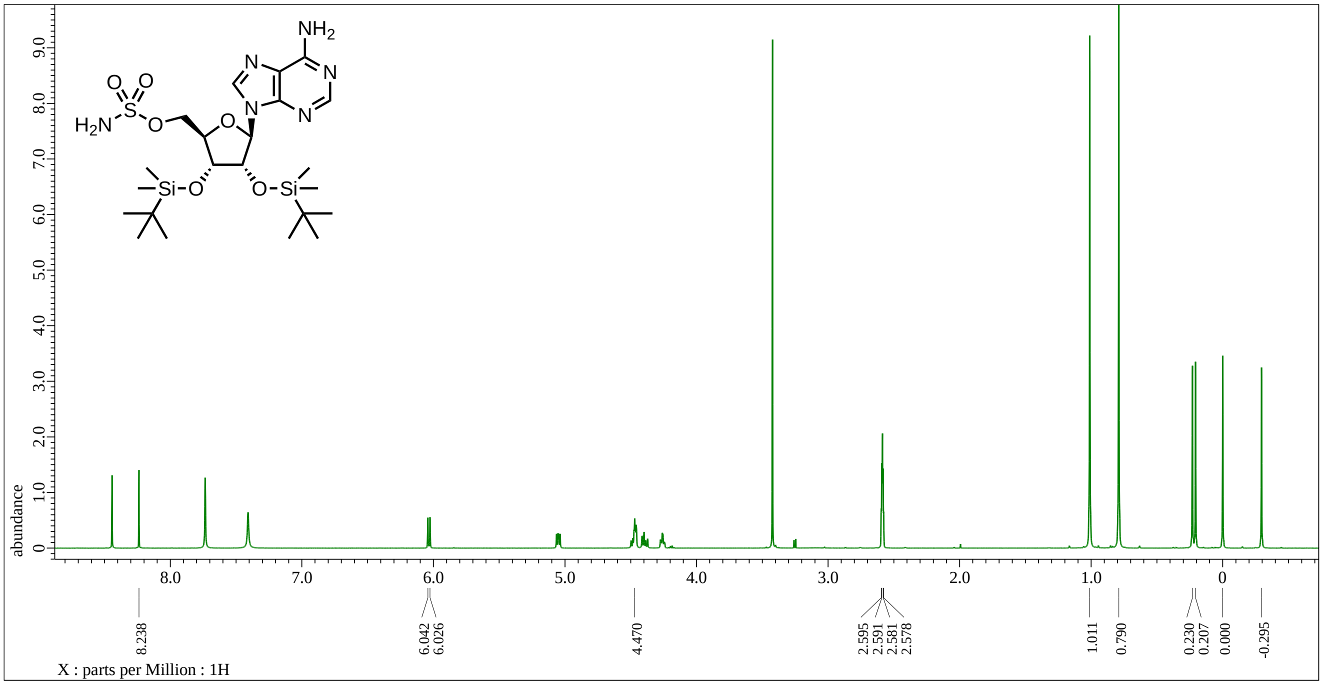

2',3'-*O,O*-Bis(*t*-butyldimethylsilyl)-5'-*O*-(*N*-decanylsulfamoyl)adenosine (6): ^{1}H-NMR ($CDCl_3$, 400MHz) δ = 8.26 (s, 1H), 5.93 (s, 1H), 4.58-4.77 (m, 3H), 4.29-4.40 (m, 2H), 2.45 (t, 2H), 1.61 (m, 2H), 1.13-1.32 (m, 12H), 0.74-0.95 (m, 2H), 0.12 (s, 3H), 0.11 (s, 3H), 0.00 (s, 3H), -0.14 (s, 3H). ESI-MS m/z: (pos) 729.39 $[M+H]^+$

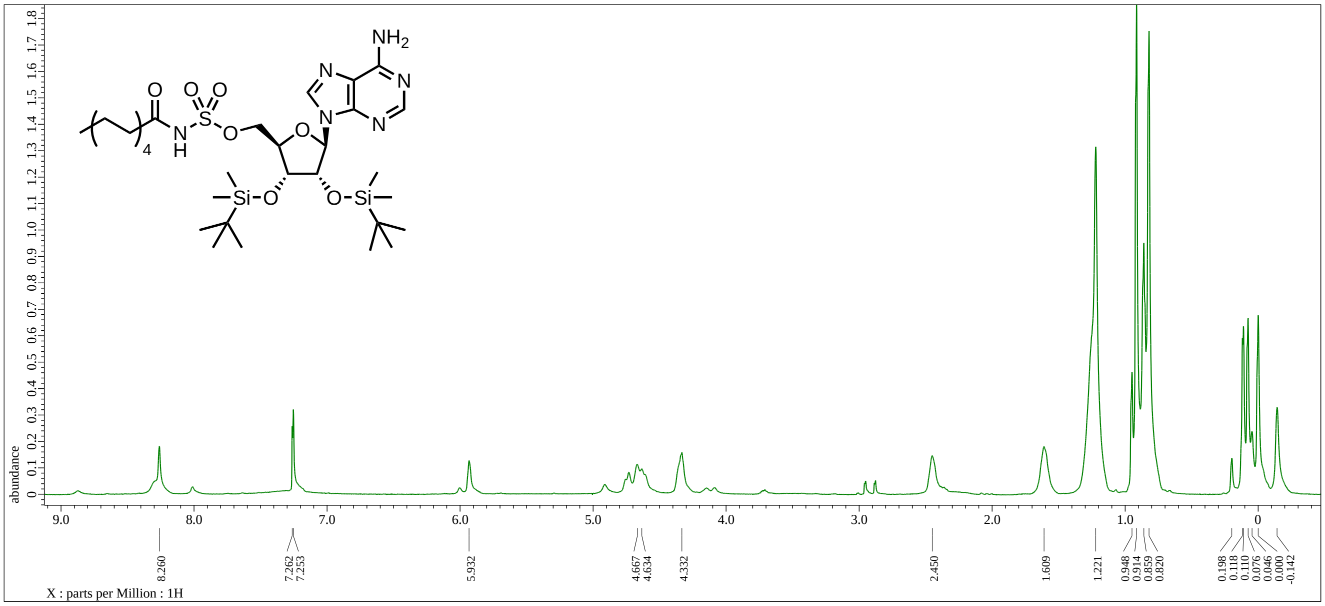

5'-*O*-(*N*-decanylsulfamoyl)adenosine mono sodium salt (C10-AMS, 7): ^{1}H-NMR (DMSOd$_6$, 400MHz) δ = 8.38 (s, 1H), 8.12 (s, 1H), 7.25-7.20 (s, 1H), 5.89 (d, 1H), 4.58 (m, 1H), 4.14. (t, 1H), 4.07 (m, 1H), 4.03 (d, 1H), 3.93-4.00 (dd, 1H), 2.49 (t, 2H), 1.96 (t, 2H), 1.15-1.23 (m, 12H), 0.82 (t, 3H). ESI-MS m/z: (pos) 501.21 $[M+H]^+$

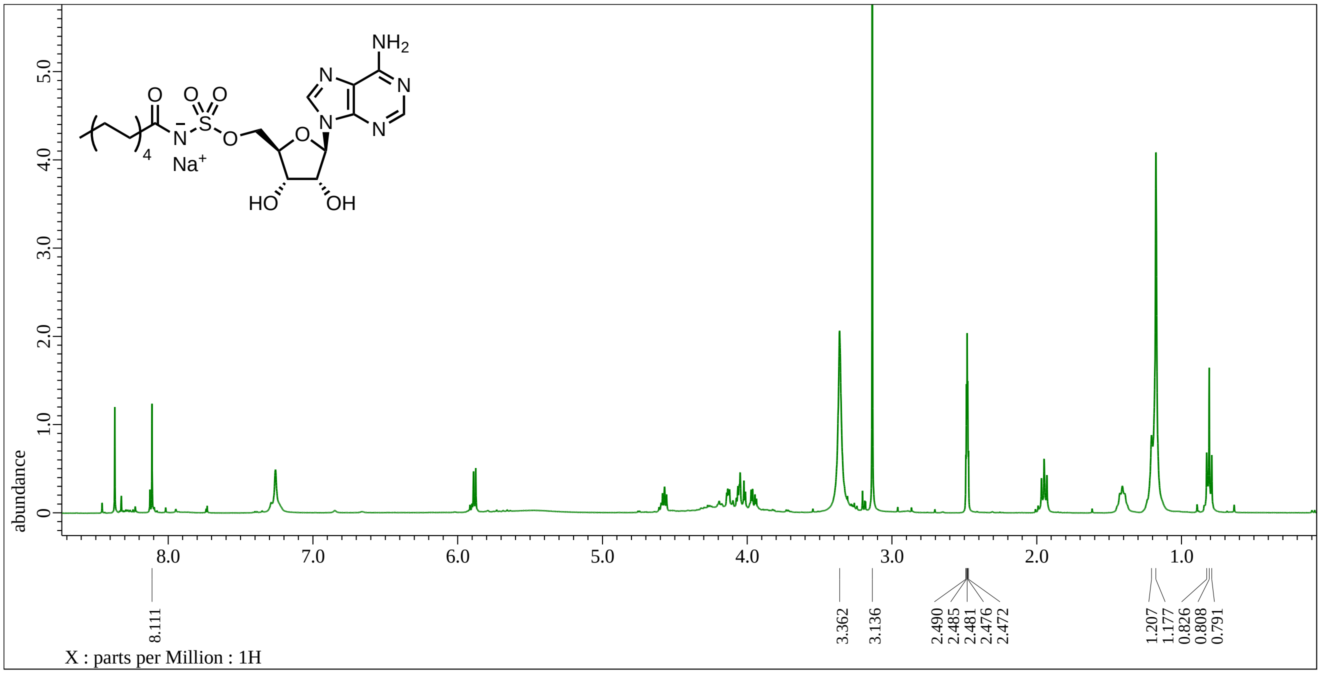

Supplement: Supplementary file 1 — Supplementary Information. [file 41598_2020_74731_MOESM1_ESM.pdf]
